# Expression of ovate family protein 8 affects epicuticular waxes accumulation in *Arabidopsis thaliana*

**DOI:** 10.1186/s40529-018-0228-8

**Published:** 2018-04-24

**Authors:** Yao Tang, Wei Zhang, Yan-Li Yin, Peng Feng, Hong-ling Li, Ying Chang

**Affiliations:** 1 Northeast Agricultural University, Harbin, 150030 China; 2 Keshan Branch of Heilongjiang Academy of Agricultural Sciences, Keshan, 160000 Heilongjiang Province China; 3 College of Pharmacy, Qiqihar Medical University, Qiqihar, 161006 China

**Keywords:** AtOFP8, Epicuticular waxes, Transcription factor

## Abstract

**Background:**

Transcription factors could regulate multiple aspects of plants growth and development, which is significant to plants. Ovate family proteins (OFPs) that are named due to contain OVATE domain, a 70-AA C-terminal conserved domain from the protein *OVATE* gene encodes, are plant-specific transcription factors family. Some members of OFPs have been shown to function as transcription factors to regulate plant growth and development, but little is known about the function of AtOFP8.

**Results:**

Here, we found AtOFP8 maybe involve in transcriptional regulation of the epicuticular waxes in *Arabidopsis thaliana*. First, we observed that the distribution of epicuticular waxes of wild type plants was more than that of *Atofp8*-*1* deletion mutants, but less than that of *35S:HA*-*AtOFP8* transgenic overexpression lines not only on the leaves but also on the stems utilizing scanning electron microscopes. Second, we extracted waxes from leaves and stems of three types of plants respectively to measure the waxes content and composition by gas chromatography–mass spectrometer (GC–MS), and the results of the total content of waxes were consistent with the results of scanning electron microscopes. Finally, we found that the expression of 12 genes related to the synthesis and metabolism of waxes was changed in the *Atofp8*-*1* mutants and *35S:HA*-*AtOFP8* transgenic lines compared with wild type plants.

**Conclusions:**

These findings suggest that AtOFP8 could change the accumulation of epicuticular waxes.

## Background

Ovate family proteins (OFPs) that are named due to contain OVATE domain, a 70-AA C-terminal conserved domain from the protein *OVATE* gene encodes, are plant-specific family of regulatory proteins (Hackbusch et al. 2005; Liu et al. 2002). All the time, we think that there are 18 genes that are predicted to encode proteins containing OVATE domain in *Arabidopsis thaliana* (Wang et al. 2007). It was not until 2014 that we knew that there are 19 OFPs genes in *A. thaliana* rather than 18 (Liu et al. 2014). *A. thaliana* ovate family proteins (AtOFPs) have been proved to be a novel transcriptional factor family, which could control multiple aspects of plant growth and development (Wang et al. 2011). So far, only a few members of AtOFPs have been shown to function as transcription factors to regulate plant growth and development (Li et al. 2011; Pagnussat et al. 2007; Wang et al. 2007). AtOFP1 was shown to regulate cell elongation by suppressing the expression of *Gibberellin 20 oxidase 1* (*GA20ox1*), a gene encoding the key enzyme in gibberellin (GA) biosynthesis (Wang et al. 2007). AtOFP5 was reported to negatively regulate the activity of a *BELL*-*like* homeodomain protein 1-*knotted1*-*like* homeodomain protein 3 (BLH1-KNAT3) complex during early embryo sac development (Pagnussat et al. 2007). AtOFP4 was demonstrated to participate in the regulation of secondary cell wall formation by interacting with KNAT7 (Li et al. 2011). However, little is known about the function of AtOFP8.

As we all know, epicuticular waxes are complex mixtures of very long chain fatty acids (VLCFAs) and their derivatives, which are significant to plants (Shepherd and Griffiths 2006). They could form an important protective layer to protect the internal structure of the plant body (Koch and Ensikat 2008). Except control the loss of water by stomatal transpiration, plants also reduce the water loss by epicuticular waxes, which plays a crucial role in resisting water stress (Sieber et al. 2000). Waxes layer on the surface of the leaves has reflection effect to ultraviolet rays and light, which can protect the leaves from the dangers of radiation (Holmes and Keiller 2002). Until now, lots of genes related to the biosynthesis and metabolism of epicuticular waxes have been reported. *LTP4*, a gene of lipid transfer proteins gene family, could bind to fatty acids and transfer phospholipids between the membranes (Arondel et al. 2000). *CER2* gene expressed in an organ- and tissue-specific manner affects epicuticular waxes accumulation and the stems of *cer2* mutants are bright green (Xia et al. 1996). *CER4* encoded an alcohol-forming fatty acyl-coenzyme A reductase (FAR) is responsible for the synthesis of primary alcohols, which involves in epicuticular waxes production (Rowland et al. 2006). *FAR4* could affect the formation of primary alcohols and the encoded enzyme could increase the length of the fatty acid chain (Domergue et al. 2010). Researchers generally believe that if stems or leaves have a glossy green phenotype, it is indicative of waxes deficiency, and conversely, if plants have whitish green stems, it is most likely displayed more waxes (Go et al. 2014).

During our cultivation of Arabidopsis, we observed that *35S:HA*-*AtOFP8* transgenic lines had whitish green leaves and *Atofp8*-*1* mutants were lighter green than wild-type plants. According to this phenomenon, we guessed the function of AtOFP8 maybe associate with the epicuticular waxes. To testify whether transcription factor AtOFP8 could affect epicuticular waxes accumulation and how to change epicuticular waxes content and composition, we had verified from multiple perspectives. In our study, we reported the difference of the shape and the content of epicuticular waxes by comparing *Atofp8*-*1* mutants, wild-type plants and *35S:HA*-*AtOFP8* transgenic lines. Moreover, the expression of 12 genes related to the biosynthesis and metabolism of waxes was significant difference among them. These results suggest transcription factor AtOFP8 could change epicuticular waxes content and composition, perhaps by changing genes expression.

## Materials and methods

### Plant materials and growth conditions

*Arabidopsis thaliana* (Columbia-0 ecotype) was used as wild-type plants in our experiment. *35S:HA*-*AtOFP8* transgenic lines and *Atofp8*-*1* mutants were previously reported (Wang et al. 2011). All seeds were planted in the 1/2 Murashige and Skoog (MS) medium containing 0.8% agar and 3% sucrose and transferred into the soil a week later. Plants were grown under long-day conditions (14 h light/10 h dark cycles) at 23 °C.

### Scanning electron microscopy

The stems and the rosette leaves of 5-week-old plants were chosen to dry in the oven at 50 °C after fixing on the foam board. Then they were plated by gold membrane on the surface of the samples glued to the sample table using ion sputtering coating machine and epicuticular waxes were observed using a scanning electron microscope.

### Transmission electron microscopy

The stems and the rosette leaves of 5-week-old plants were chosen to fix in a solution containing 2.5% glutaraldehyde in 0.1 mol L^−1^ phosphate buffer, pH 6.8, at 4 °C for 4 h. The samples were then rinsed in 0.1 mol L^−1^ phosphate buffer, pH 6.8, three times and further fixed in 1% osmium tetroxide for 3 h at 4 °C. After rinsing with 0.1 mol L^−1^ phosphate buffer at normal temperature, the samples were dehydrated and embedded in Spurr’s resin. After processing embedded block, thin sections (60 nm thickness) were prepared with an ultramicrotome. After staining, the sections were observed under a transmission electron microscope.

### GC–MS analysis

150 mg of the stems and the rosette leaves of 5-week-old plants were weight to put into small beakers respectively and 5 mL of chloroform were added into small beakers for 1 min at room temperature. After adding 5 µg of C24 into the extracted chloroform solvent as internal reference, the solvent was transferred into GC bottles and evaporated under a gentle stream of nitrogen. Then 10 µL bis(trimethylsilyl)trifluoroacetamide (BSTFA) and 10 µL pyridine were added into the GC bottles to redissolve waxes samples and the waxes mixtures were heated for 1 h at 70 °C. Samples were measured by injecting 1 µL of sample and helium (2 mL min^−1^) was used as a carrier gas during which the oven temperature was increased from 80 to 290 °C at a rate of 4 °C/min and finally 30 min at 290 °C.

### Gene chip analysis

When the plants grew up to 3 weeks old, leaves were cut off and sent to Shanghai Bohao Biotechnology Corporation to make agilent chip to find differentially expressed genes. All chip results were analyzed by SBC analysis system (SAS), an expression microarray data analysis software of this corporation.

### Quantitative real-time RT-PCR analysis

RNA of 3-week-old plants leaves was extracted using RNA extraction kit according to the manufacturer’s instructions. Reverse transcription (RT) was performed according to reverse transcription kit instructions to synthesize first-strand cDNA. Quantitative RT-PCR reactions were performed using SYBR green fluorescent quantitation PCR kit. The PCR primers used were listed in Table 1. The values for each set of primers were normalized relative to the *ACTIN 2* gene. qRT-PCR reactions were performed in biological triplicates using RNA samples extracted from three independent replicate samples. The comparative ∆∆C_T_ method was used for the calculation of the results.

**Table 1:** qRT-PCR primer sequence

Gene	Primer name	Primer sequence (5′–3′)
LTP4	LTP4-F	CACCTCCGTGCTGTGCAG
	LTP4-R	TGGCGCAGTTGGTGCTC
At2g18370	At2g18370-F	GCATCAAGTCAGTGGCTAATAGTGT
	At2g18370-R	CATTCGCCTACATATATTATACGGC
CER4	CER4-F	TTGATGCTGTTTCTGATGTGATGC
	CER4-R	ACGACCTTCCCTTCTTTGTTGG
KCS2	KCS2-F	GGTTGTAGTGCTGGTCTTATCTCC
	KCS2-R	GGACGGTGTGGATGAGTTGG
ADH1	ADH1-F	ATGCCTTCAAGACTCATCCGA
	ADH1-R	TTGATTTCCGAGAATGGCACT
KCS10	KCS10-F	CTCGACGTCAGTTTAATGCATCT
	KCS10-R	GGGTTCTTGGTAGCGTATTCGT
FAR4	FAR4-F	CACAATGGCTCAGTTCAGTTTCTAC
	FAR4-R	CGCAGTTTCTCAGTATTCGTATCG
GL2	GL2-F	TAGAGATGAAGCTCGTCGGCAT
	GL2-R	GTTGCCTCTGTCTTGTCCCTT
DFR	DFR-F	GGTTACTTTGTTCGTGCCACC
	DFR-R	CCTCAGATAAATCAGCCTTCC
At2g42990	At2g42990-F	GCCTCGAAAAGATGTGAAGAG
	At2g42990-R	TGAATTCTTAACACGCCAGAGG
At1g06360	At1g06360-F	GACCCACATAGCCCTATCGAA
	At1g06360-R	TGCTTCAAGTCCATCACGTT
CER2	CER2-F	CGAGCCGTCTACTTCTTCAAGG
	CER2-R	GTTGCAGCGAATGTAAGGTATGG
ACT2	ACT2-F	CCAGAAGGATGCATATGTTGGTGA
	ACT2-R	GAGGAGCCTCGGTAAGAAGA

### Chlorophyll leaching assays

When the plants grew up to 5 weeks old, 1 g of rosette leaves was weight and put into erlenmeyer flasks wrapped with aluminum foil. Then 30 mL of 80% ethanol were poured into erlenmeyer flasks. Remove 3 mL solution from erlenmeyer flasks to measure absorbance at 647 and 664 nm using a spectrophotometer as the amount of extracted chlorophylls every 10 min. The solution using for measuring was back into erlenmeyer flasks every time. The absorbance measured after 24 h was as the total amount of chlorophylls. The calculation of chlorophyll content was based on the method previously (Lolle et al. 1998).

### Water loss assays

The rosette leaves of 5-week-old plants were cut out and put into water for 1 h in the dark to make water contents be same. After drying the water on the surface of leaves, the leaves were weight using a microbalance every 20 min. The water loss rate (v) was calculated by the formula:

$${\text{V}} = {{\left( {{\text{W}}_{0} {-}{\text{W}}_{ 1} } \right)} \mathord{\left/ {\vphantom {{\left( {{\text{W}}_{0} {-}{\text{W}}_{ 1} } \right)} {{\text{W}}_{0} }}} \right. \kern-0pt} {{\text{W}}_{0} }} \times 100\%$$

Where W_0_, weight of leaves before dehydration; W_1_, weight of leaves after dehydration.

## Results

### Identification of plant materials

*35S:HA*-*AtOFP8* transgenic plants and *Atofp8*-*1* mutant were previously reported (Wang et al. 2011). Gene chip results showed that the transcription level of *AtOFP8* in *35S:HA*-*AtOFP8* overexpression plants increased more than 300-fold, while decreased by nearly 14-fold in the *Atofp8*-*1* mutant (Table 2).

**Table 2:** The transcription level of *AtOFP8* in *35S:HA*-*AtOFP8* overexpression plants and *Atofp8*-*1* mutant

Locus	Symbol	Accession	FC in *35S:HA*-*AtOFP8*	FC in *Atofp8*-*1*
AT5G19650	OFP8	NC_003076	323.1614	− 13.7830

### Leaves are whitish green in *35S:HA*-*AtOFP8*

**Fig. 1:**
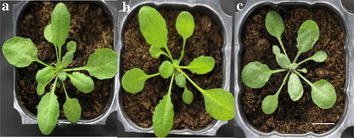
Image of seedlings 25 days old. **a** Col-0, **b** *Atofp8*-*1*, **c** *35S:HA*-*AtOFP8*. Bar = 1 cm

After transplanting plants in the soil, we gradually found there were significant differences in the color and phenotype of leaves among them. Although there was no significant difference in the phenotype of leaves between *Atofp8*-*1* and Col-0, leaves of *35S:HA*-*AtOFP8* were thicker and flatter than that of Col-0. Moreover, leaves of *35S:HA*-*AtOFP8* were more whitish green than that of Col-0, whereas leaves in *Atofp8*-*1* were lighter green than that of Col-0 (Fig. 1). We guessed this phenomenon might be related to epicuticular waxes.

### The distribution of epicuticular waxes is thin in *Atofp8*-*1*, whereas dense in *35S:HA*-*AtOFP8*

**Fig. 2:**
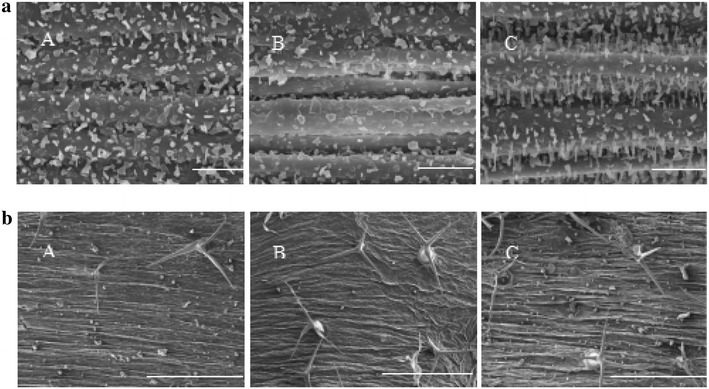
Scanning electron microscopy images of epicuticular waxes. **a** Images of the stems in the Col-0 (A), *Atofp8*-*1* (B) and *35S:HA*-*AtOFP8* (C). Bars = 10 µm. **b** Images of the leaves in the Col-0 (A), *Atofp8*-*1* (B) and *35S:HA*-*AtOFP8* (C). Bars = 10 µm

**Fig. 3:**
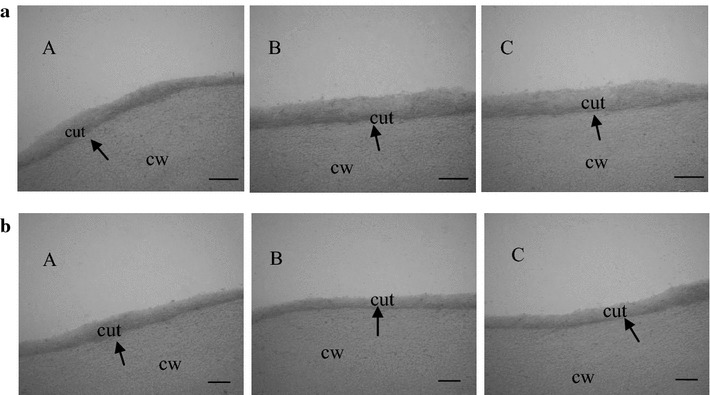
Transmission electron microscopy images of epicuticular waxes. *cut* cuticle, *cw* cell wall. **a** Images of the stems in the Col-0 (A), *Atofp8*-*1* (B) and *35S:HA*-*AtOFP8* (C). Bars = 200 nm. **b** Images of the leaves in the Col-0 (A), *Atofp8*-*1* (B) and *35S:HA*-*AtOFP8* (C). Bars = 200 nm

Depending on the difference in the color and phenotype of leaves, we guessed this was most likely caused by the different distribution of epicuticular waxes. In order to verify whether this was related to the distribution of epicuticular waxes, surface of stems and leaves were observed respectively using the scanning electron microscope. On the surface of stems, the morphology of epicuticular waxes in *Atofp8*-*1*, Col-0 and *35S:HA*-*AtOFP8* are clearly different each other. Moreover, epicuticular waxes in Col-0 seem to lie more densely than that in *Atofp8*-*1*, but not so densely as the *35S:HA*-*AtOFP8* (Fig. 2a). Changes in content were the same in the leaves (Fig. 2b). Then we did transmission electron microscopy analysis to leaves and stems of *Atofp8*-*1*, Col-0 and *35S:HA*-*AtOFP8*. The results showed that there were no significant differences in the thickness of the cuticle in neither leaves nor stems (Fig. 3a, b). These observations imply that AtOFP8 conld only affect the distribution of epicuticular waxes without changing the cuticle waxes.

### The content of waxes is reduced in *Atofp8*-*1*, while increased in *35S:HA*-*AtOFP8*

**Fig. 4:**
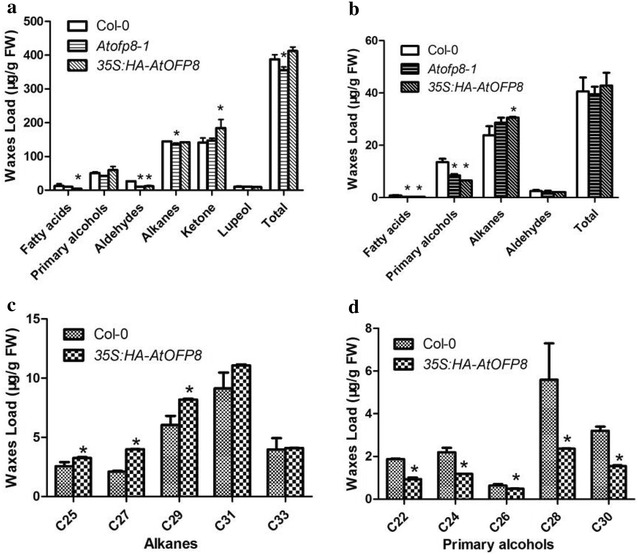
Epicuticular waxes accumulation in the stems (**a**) and leaves (**b**–**d**). 5 weeks old stems and leaves were used. Biological triplicates, each with technical duplicates, were averaged. Statistical significance of the measurements was by comparing Col-0 (*P < 0.05). Bars indicate SE of the mean. *FW* fresh weight

To verify further whether waxes content was changed, GC-MS was used to measure the content in the stems and leaves respectively. Although the content of waxes was not changed exponentially, there was still some difference among the totals of waxes. Results showed that the totals of waxes were 387.56, 356.05 and 412.23 µg g^−1^ respectively in the stems of Col-0, *Atofp8*-*1* and *35S:HA*-*AtOFP8*. Compared with the wild type plants, the total content of waxes of *Atofp8*-*1* was slightly lower, while the total content of waxes in *35S:HA*-*AtOFP8* transgenic plants was slightly higher (Fig. 4a). The different components of the stems waxes also changed. The content of primary alcohols and ketone in *35S:HA*-*AtOFP8* transgenic plants was significantly higher than that in wild type, while *Atofp8*-*1* did not change significantly with wild type (Fig. 4a). However, the totals of waxes in the leaves were almost approximate (Fig. 4b), but there were distinct variations between the specific components. In the leaves of the *35S:HA*-*AtOFP8*, alkanes were significantly increased compared with the wild type plants, while primary alcohols were significantly reduced (Fig. 4c, d). These results indicate that AtOFP8 could change the content and composition of waxes.

### Gene chip results show that AtOFP8 affects many aspects of Arabidopsis growth and development

**Fig. 5:**
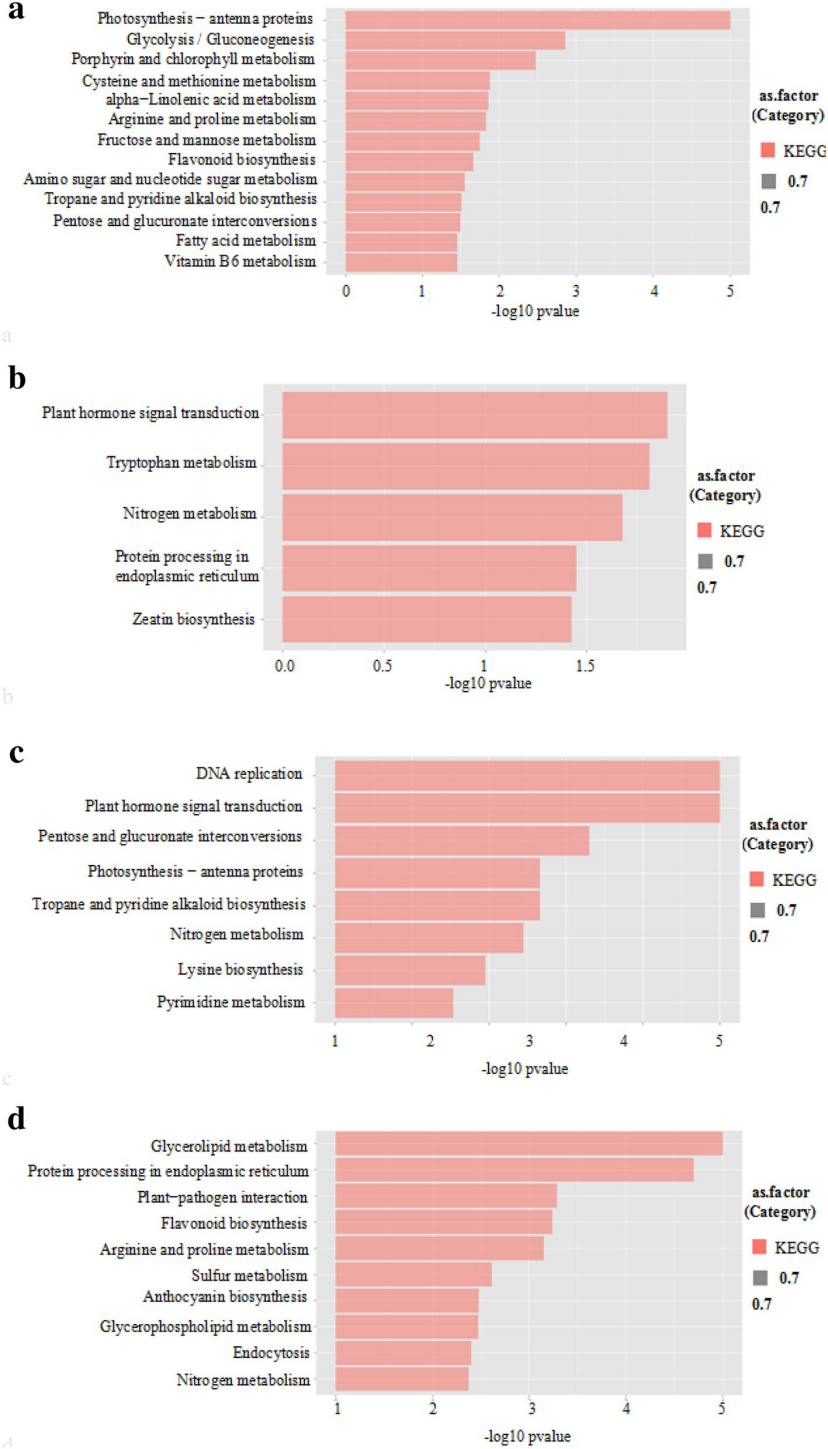
Pathway enrichment affected by AtOFP8 gene. **a** Pathway enrichment of *35S:HA*-*AtOFP8* vs Col-0 up-regulated genes. **b** Pathway enrichment of *35S:HA*-*AtOFP8* vs Col-0 down-regulated genes. **c** Pathway enrichment of *Atofp8*-*1* vs Col-0 up-regulated genes. **d** Pathway enrichment of *Atofp8*-*1* vs Col-0 down-regulated genes

To find out which genes caused by the change of epicuticular waxes, we made gene chip analysis among the *Atofp8*-*1*, *35S:HA*-*AtOFP8* and Col-0. Results showed that the expression of abundant genes was changed in the *Atofp8*-*1* and *35S:HA*-*AtOFP8* compared with Col-0. FC (*35S:HA*-*AtOFP8* vs Col-0) represented the ratio of gene expression level in *35S:HA*-*AtOFP8* transgenic plants to Col-0 in the data analysis. FC (*Atofp8*-*1* vs Col-0) represented the gene in *Atofp8*-*1* mutant and Col-0 expression levels in the ratio. It was generally considered that there was no significant difference in gene expression in the range of 0.5–2.0 for FC values, while genes with FC values greater than 2 or less than 0.5 were considered significant expression differences. There were 300 genes whose FC (*35S:HA*-*AtOFP8* vs Col-0) value was more than 2 or less than 0.5, among which 192 genes were up-regulated, which were mainly related to photosynthesis, glucose metabolism, amino acid metabolism, fatty acid metabolism, alkanes and flavonoids biosynthesis (Fig. 5a). 108 genes were down-regulated, and these genes were associated with plant hormone signal transduction, tryptophan metabolism, nitrogen metabolism, endoplasmic reticulum protein processing and zeatin biosynthesis (Fig. 5b). There were 434 genes with FC (*Atofp8*-*1* vs Col-0) value more than 2 or less than 0.5, which 268 genes were up-regulated, mainly related to DNA replication, plant hormone signal transduction, glucose metabolism, photosynthesis, alkanes, pyrimidines and organisms alkaloid biosynthesis processes (Fig. 5c). 166 down-regulated genes were mainly associated with processes such as glyceride metabolism, endoplasmic reticulum protein processing, plant pathogen interactions, flavonoid biosynthesis, amino acid metabolism, and anthocyanin synthesis (Fig. 5d).

### The expression of genes related to epicuticular waxes varies greatly in *35S:HA*-*AtOFP8*

**Fig. 6:**
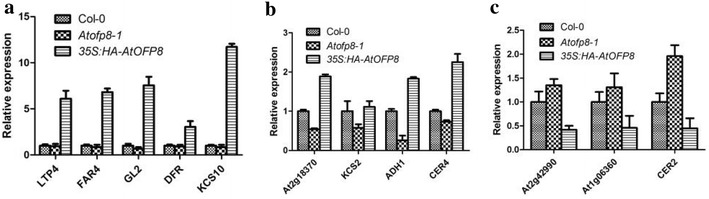
Expression of genes that relate to waxes in Arabidopsis (**a**, **b**, **c**). The leaves of 3 weeks old plants were used. Bars indicate SE of the mean

We chose some genes involved in epicuticular waxes synthesis and metabolism from gene chip results to test by qRT-PCR (Table 3). Results showed that the expression of *KCS10* was up-regulated by more than tenfold in *35S:HA*-*AtOFP8* compared with Col-0. The expression of *LTP4*, *FAR4* and *GL2* also increased by more than 5 times in *35S:HA*-*AtOFP8* (Fig. 6a). In addition, the expression of *CER4*, *ADH1* and *At2g18370* was raised about twice in *35S:HA*-*AtOFP8*, while significantly down-regulated in *Atofp8*-*1* compared with Col-0 (Fig. 6b). Contrary to these, the expression of *CER2*, *At2g42990* and *At1g06360* in *35S:HA*-*AtOFP8* was less than half of Col-0, while up-regulated in *Atofp8*-*1* compared with Col-0 (Fig. 6c). These results show that AtOFP8 could change the expression of genes involving in epicuticular waxes accumulation, largely promoting the expression.

**Table 3:** Genes about epicuticular waxes accumulation

Gene	Symbol	FC in *35S:HA*-*AtOFP8*	FC in *Atofp8*-*1*	Description
At5g59310	LTP4	4.6633	1.1343	Lipid transfer protein 4
At2g18370		2.2816	− 1.7933	Lipid transfer protein
At4g33790	CER4	1.9469	− 1.2743	Fatty acyl-CoA reductase
At1g04220	KCS2	1.9174	− 1.0575	3-Ketoacyl-CoA synthase 2
At1g77120	ADH1	1.9057	− 2.2203	Alcohol dehydrogenase 1
At2g26250	KCS10	1.8824	− 1.2770	3-Ketoacyl-CoA synthase 10
At3g44540	FAR4	1.8416	− 1.2542	Fatty acyl-CoA reductase 4
At1g79840	GL2	1.6498	− 1.3211	Lipid binding function
At5g42800	DFR	1.4015	− 2.5641	Dihydroflavonol 4-reductase
At2g42990		− 1.0988	2.9812	Lipid catabolic process
At1g06360		− 1.2762	1.7454	Fatty acid desaturase family protein
At4g24510	CER2	− 1.1792	1.4884	Fatty acid elongation

### Cuticle transpiration slows in *35S:HA*-*AtOFP8*

**Fig. 7:**
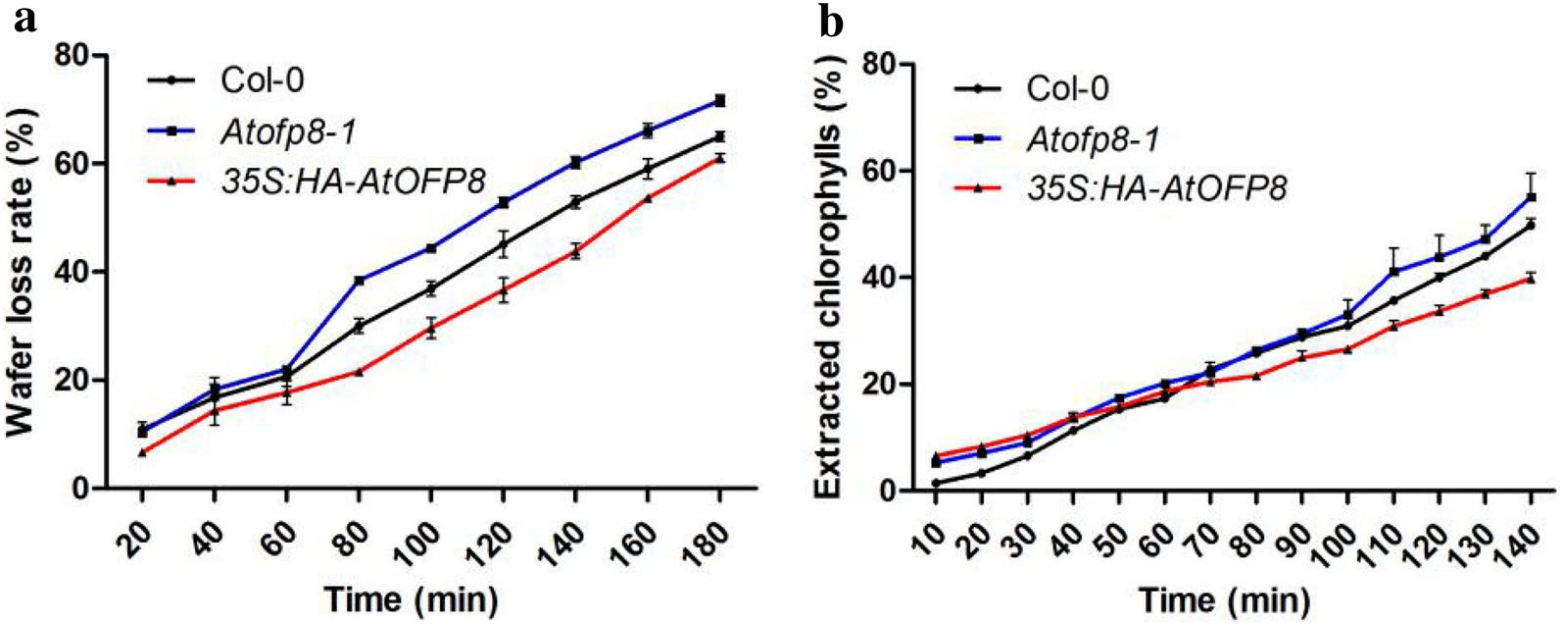
Cuticular transpiration in Col-0, *Atofp8*-*1* and *35S:HA*-*AtOFP8* leaves. 5 weeks old leaves were used. Bars indicate SE of the mean. **a** Water loss assays. **b** Chlorophyll leaching assays

After observing so many changes related to epicuticular waxes, we made water loss assays to test whether stratum corneum changed. Results showed that water loss in Col-0 was slower than that in *Atofp8*-*1*, but faster than that in *35S:HA*-*AtOFP8* (Fig. 7a). Further chlorophyll leaching experiment showed that the percentage is the lowest in *35S:HA*-*AtOFP8* after 70 min (Fig. 7b). These results indirectly prove that there are more epicuticular waxes distributed on the leaves surface of *35S:HA*-*AtOFP8*.

## Discussion

To explore the function of AtOFP8, we designed this experiment using *Atofp8*-*1*, Col-0 and *35S:HA*-*AtOFP8.* We analyzed microscopic distribution of epicuticular waxes, all kinds of component content, gene expression difference, which all results could demonstrate that AtOFP8 was able to regulate epicuticular waxes accumulation in some way. This laboratory finding provides a new direction for research AtOFP8 even AtOFPs.

Although the total amount of waxes in leaves was little difference, changes in specific components were obvious. Maybe AtOFP8 only changed the conversion of some certain components in leaves, so the total amount of waxes in leaves changed little. For example, in the *35S:HA*-*AtOFP8* leaves, alkanes were significantly increased compared with the wild type plants, while primary alcohols were significantly reduced (Fig. 4c, d). Another reason was the presence of functional redundancy. Loss of AtOFP8 might induce expression of other genes that had the same function, so the content of epicuticular waxes in the *Atofp8*-*1* did not change significantly.

Due to diverse component of epicuticular waxes and its complex metabolic process, we do not determine what a role AtOFP8 plays in the metabolic process of waxes. In addition, functional redundancy exists in the transcription factors AtOFPs all the time, so it is difficult to find the its specific function invisibly. This is another reason that we cannot understand which part AtOFP8 affects epicuticular waxes accumulation. In spite of these, various aspects of changes of epicuticular waxes are obvious enough to prove that it plays an important role that AtOFP8 could change genes expression to affect epicuticular waxes accumulation.

## Abbreviations

AtOFP: *Arabidopsis thaliana* ovate family proteins
BSTFA: bis(trimethylsilyl)trifluoroacetamide
GC–MS: gas chromatography–mass spectrometer
MS: Murashige and Skoog
OFPs: ovate family proteins
SAS: SBC analysis system
VLCFAs: very long chain fatty acids
